# Cyclic flexural fatigue resistance of NiTi Controlled Memory and Blue Technology instruments after torsional preloading

**DOI:** 10.1590/1678-7757-2018-0144

**Published:** 2018-08-20

**Authors:** Túlio César MODESTO, Eufemia Carolina Peláez ACOSTA, Pedro Damas RESENDE, Érika Sales Joviano PEREIRA, Isabella Faria da Cunha PEIXOTO, Vicente Tadeu Lopes BUONO, Ana Cecília Diniz VIANA

**Affiliations:** 1 Universidade Federal de Minas Gerais, Faculdade de Odontologia, Departmento de Odontologia Restauradora, Belo Horizonte, MG, Brasil.; 2 Universidade Federal de Minas Gerais, Faculdade de Engenharia, Departmento de Engenharia Metalúrgica e de Materiais, Belo Horizonte, MG, Brasil.; 3 Universidade Federal da Bahia, Faculdade de Odontologia, Departamento de Clínica Odontológica, Salvador, BA, Brasil.

**Keywords:** Endodontics, Dental Instruments, NiTi

## Abstract

**Objective:**

The aim of this study was to evaluate the influence of torsional preloading on the cyclic flexural fatigue resistance of thermally treated NiTi instruments.

**Material and Methods:**

Ten new instruments New Hyflex CM (HF 30.06; Coltene/Whaladent Inc.), Typhoon CM (TYP 30.06; Clinician's Choice Dental Products) and Vortex Blue (VB 30.06; Dentsply Tulsa Dental) were chosen, based on geometry and specific characteristics of the manufacturing process. The new instruments of each system were tested in a bench device to determine their fatigue resistance through mean value of number of cycles to failure (Nf) (Control Group – CG). Another group of 10 new HF, TYP and VB instruments were submitted to 20 cycles of torsional straining between 0° and 180° (Experimental Group – EG) and then submitted to fatigue until rupture under the same conditions of the CG. Tested instruments were examined by scanning electron microscopy (SEM). Data were analyzed using one-way analysis of variance and post hoc Tukey’s test (α=.05).

**Results:**

Higher fatigue resistance was accomplished by HF instruments, followed by VB and TYP (p<0.05). During the torsional preloading, the lowest mean torque value was observed for TYP instruments (p<0.05). The torsional preload caused a significant reduction in the Nf values (p<0.05) of about 20%, 39% and 45% for instruments HF, VB and TYP, respectively. Longitudinal cracks, generated during the torsional preloading, were present in VB files, but were not observed in the CM instruments (HF and TYP).

**Conclusions:**

In conclusion, the flexural fatigue resistance of thermally treated instruments is diminished after cyclic torsional loading.

## Introduction

Thermomechanical manufacturing technologies have been developed to improve the mechanical properties of NiTi endodontic instruments. This approach allows adjusting transition temperatures of the NiTi alloy, aiming to control its microstructure and thus influence the mechanical behavior of the instruments.^1^ The final goal of these specific thermal treatments is to produce instruments with higher flexibility and increased resistance to cyclic flexural fatigue.^2^ ^,^ ^3^

Controlled Memory (CM) instruments such as Hyflex CM (HF; Coltene/ Whaladent Inc; Cuyahoga Falls, OH, USA) and Typhoon CM, (TYP; Clinician’s Choice Dental Products; New Milford, CT, USA) represent the latest generation of rotary shape memory NiTi files. According to the manufacturers, because of the chemical compositions and specific manufacturing processes employed, CM instruments are not superelastic, they do not recover their original shape after tension removal.^4^ ^-^ ^6^ Another instrument that receives a proprietary thermomechanical treatment is Vortex Blue rotary file (VB, Dentsply Tulsa Dental Specialties; Tulsa, OK, USA). These instruments present a particular blue color, not commonly seen in the traditional superelastic NiTi files, because of a visible titanium oxide layer formed during thermal treatment.^1^ ^,^ ^2^ ^,^ ^7^ ^,^ ^8^

In the clinical practice, fractures may happen under many circumstances, being more common during shaping curved and/or narrow root canals. Torsion, compression and tensile strains take place simultaneously during the canal preparation. Even so, most of the studies evaluate the fracture behavior of NiTi files separately.^9^ ^,^ ^10^ Therefore, by combining different straining modes, we could have results that better simulate the clinical process.

Nevertheless, torsional cycling decreased the cyclic flexural fatigue resistance of superelastic NiTi files, probably because of the generation of longitudinal cracks during cyclic loading.^11^ How thermal-treated NiTi instruments would behave when submitted to this kind of complex multiaxial loading it is not very clear yet. Cyclic flexural fatigue and torsional resistance of NiTi files have been extensively evaluated as isolated processes for superelastic files.^12^ ^-^ ^14^ However, a few reports assessing these factors at the same time are available for CM instruments.

Therefore, the aim of this study was to evaluate the influence of torsional preloading on the cyclic flexural fatigue resistance of the thermal-treated NiTi instruments HF, TYP, and VB to simulate active loads on the instrument as close as possible to the clinical reality. The null hypothesis is that torsional preloading does not influence the cyclic flexural fatigue resistance of the NiTi endodontic instruments.

## Materials and methods

A total of 60 new Hyflex CM, Typhoon CM and Vortex Blue 30/.06 files were used in this study. TYP and VB files present similar geometric designs, with a convex triangular cross section, whereas HF instruments display a triangular cross section.

Ten new instruments of each system were photographed using a high-resolution digital camera Canon EOS 20D (Canon Inc; Tokyo, Kanto; JP) to evaluate their dimensional characteristics such as diameter at 3 mm from the tip (D3), based on the American National Standards Institute/American Dental Association Specification No. 101. The software Image J 1.49 V (National Institute of Health NIH; Bethesda, MD, USA) was employed for the measurements. The cross-sectional area of instruments sectioned at 3 mm from the tip (A3) was also evaluated through scanning electron microscopy images (SEM) Inspect F50 (FEI^®^; Hillsboro, OR, USA) using the same software.

The files were divided into two groups: (i) a Control Group (CG), comprising 10 instruments of each system, which were tested in cyclic flexural fatigue to determine the average number of cycles to failure (Nf) of new instruments; and (ii) an Experimental Group (EG), where 10 instruments of each system were submitted to 20 torsion cycles from 0° to 180° and then submitted to the cyclic flexural fatigue test until rupture.

The cyclic flexural fatigue tests were performed in a room under constant temperature of 25°C in a bench device described earlier.^15^ The files were stabilized inside an artificial canal for a locking system, standardizing their positions. When the test started, they rotated freely inside an artificial canal made up of AISI H13 tool steel, consisting of an arch whose angle of curvature was 45°, with a radius of 5 mm and a guide cylinder of 10 mm in diameter, made of the same material. To ensure accurate time of instrument rupture the test was performed under a stereomicroscope with a digital camera KL200 (Carl Zeiss; Göttingen, Niedersachsen, GER) at ×10 magnification.

The selected geometry placed the maximum curvature at 3 mm from the tip.^15^ The files rotated freely inside the canal until rupture, and the Nf was calculated by multiplying the rotation speed by the time registered using a digital chronometer (Prolab^®^; São Paulo, SP, BRA). During the experiment, a mineral oil (Castrol™; Brisbane, Queensland, AUS) was used between each instrument inside the artificial canal to prevent wear of the instruments caused by friction. The rupture point was measured by decreasing the total length of the instrument by its final length after rupture with a quality endodontic ruler (Dentsply Maillefer; Balaigues, Vaud, SWI) under a stereomicroscope with a digital camera KL200 (Carl Zeiss; Göttingen, Niedersachsen, GER) at ×10 magnification.

The torsion tests were performed in an apparatus built based on the International Organization for Standardization ISO 3630-1 AN8050 (Analógica; Belo Horizonte, Minas Gerais, BRA). The rotation speed was set clockwise to 2 rpm. The handle was clamped into an appropriate holder and three millimeters of the instrument tip were clipped to a brass jaw to prevent sliding. Continuous recording of torque and angular deflection were provided by specific designed computer program. The machine was programmed to repeatedly perform 20 cycles of torsional loading from 0° angular deflection to 180° and then rotate back to zero applied torque. Each rotation was defined as one cycle. Ten new files of each system were subjected to 20 cycles of torsional preloads, as described. Subsequently, these instruments were tested in cyclic flexural fatigue until rupture, under the same conditions aforementioned. The number of cycles employed in the load–unload tests was selected based on the assumption that an instrument requires an average of three strokes to shape one root canal.^16^ Thus, 20 torsional strain cycles should be roughly equivalent to the use of the instrument in six root canals, which represents approximately half of the recommended number of uses for conventional NiTi rotary instruments.^12^

Cycled instruments were observed by SEM before and after rupture in cyclic fatigue. Results of the tests performed were analyzed using one-way analysis of variance ANOVA, and *post hoc* Tukey’s test at a significance level of *P*>0.05.

## Results

The mean values of the diameter at three millimeters from the tip (D3) of the analyzed instruments (HF, TYP and VB) were equal to 0.48±0.01 mm, with no statistical significant differences among instruments (*P*>0.05). The instruments with triangular convex cross-section (VB and TYP) showed larger average values of cross-sectional area A3 such as 0.128 mm^2^ and 0.104 mm^2^ in comparison with the HF instruments, which were 0.089 mm^2^, with triangular cross-sectional area. The average values of A3 for the three instruments were statistically different (*P*<0.05).

**Figure 1:**
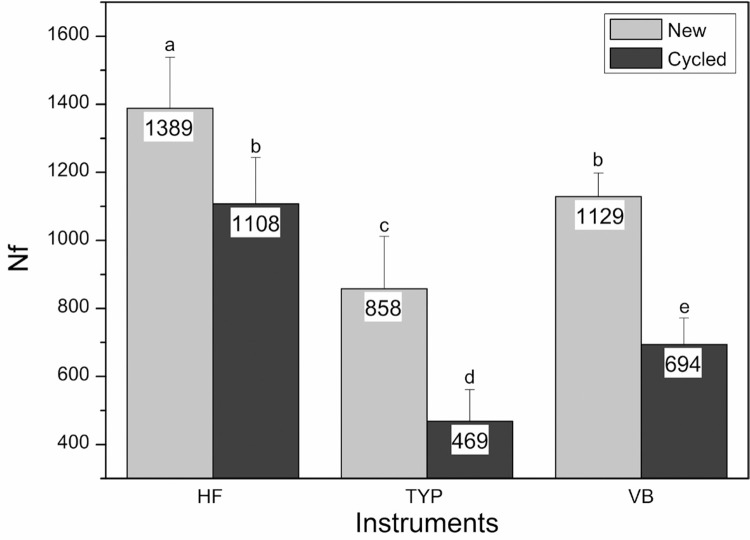
The mean Nf values measured in the cyclic flexural fatigue test for HF, VB and TYP instruments. New and preloaded instruments. Standard deviations (SD) are shown as error bars. Differently labeled columns represent statistically significant differences (p≤0.05)

Figure 1 summarizes the results of the cyclic flexural fatigue tests. Among new instruments (CG), HF exhibited the highest Nf value (1389±149), followed by VB (1129±69), and TYP (858±154) instruments. The mean Nf values of the instruments previously submitted to 20 cycles of torsion from 0° to 180° (EG) are also shown in Figure 1. Statistically significant differences were observed among all of the new and preloaded instruments (*P*<0.05). Statistical analysis comparing each type of instrument in CG and EG showed significant statistical differences for all the files (*P*<0.05), indicating that torsional straining diminished the cyclic flexural fatigue resistance of the instruments. There were average reductions of 20%, 39% and 45% of the cyclic flexural fatigue resistance for the instruments HF, VB and TYP, respectively. The location of the fracture was about 3 mm (±.02) for all instruments analyzed, with no statistical significant differences among them.

**Figure 2:**
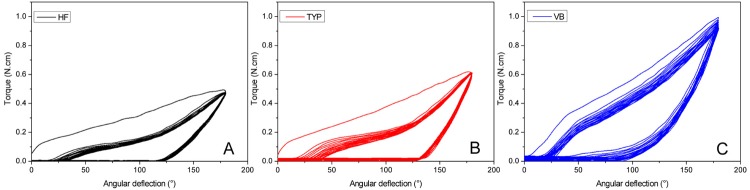
Typical torsional loading curves of instruments HF(A), TYP(B) and VB(C) submitted to 20 loading cycles

Figure 2 (A-C) illustrates the typical torsional loading curves. The lowest mean torque value at 180° was observed for the HF instruments (0.562 N.cm) followed by TYP (0.667 N.cm) and VB (0.980 N.cm).

Angular deflection values remaining when the torque reaches zero after unloading were higher for HF and TYP (124° and 130°, respectively), which were statistically significant in relation to VB (90°) instruments (*P*<0.05).

**Figure 3:**
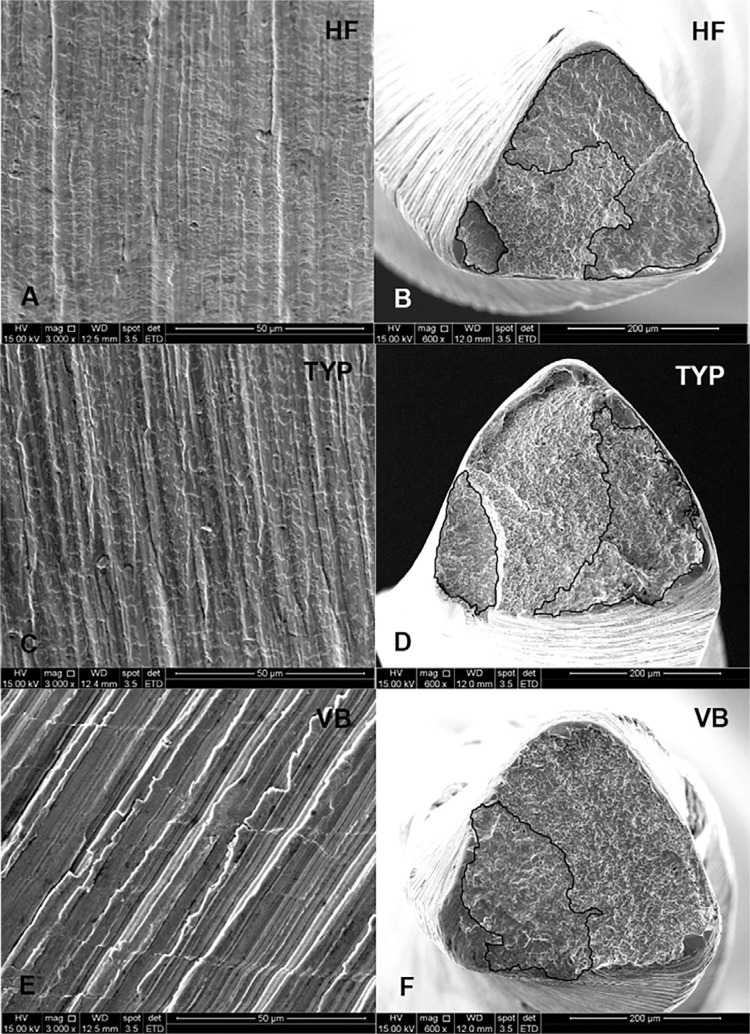
Scanning electron microscopy images of typical lateral surface of cyclic strained instruments (A=Hyflex, C=Typhoon, E=Vortex Blue) and from surfaces that suffered rupture (B=Hyflex, D=Typhoon, F=Vortex Blue) after the cyclic flexural fatigue test (outlined areas correspond to the region of fatigue crack nucleation and propagation)

Figure 3 (A-F) shows the scanning electron microscopy images taken from the lateral surface of cyclic strained instruments and from surfaces that suffered rupture after the cyclic flexural fatigue test. The lateral images of the CM instruments (Figures 3A and 3C) showed extensive fragmentation of the oxide layer during straining, which was not observed on the VB instrument (Figure 3E). Nevertheless, the expected presence of longitudinal cracks was only noticed in the latter. The typical features of cyclic flexural fatigue rupture can be seen in the fracture surfaces of the instruments: the smooth regions of crack nucleation and slow propagation (outlined) and the areas of final ductile rupture. Larger areas of crack nucleation and slow propagation were found in the HF instruments (Figure 3B), in comparison with TYP (Figure 3D) and VB ones (Figure 3F).

## Discussion

Thermal, mechanical or a combination of both treatments have been proposed and applied to the NiTi wire and/or endodontic instrument, considerably enhancing their flexibility and cyclic flexural fatigue resistance compared with instruments made of conventional alloy.^1^ ^,^ ^4^

It has been reported in the literature that CM instruments contain a great amount of B19` and R-phase at room temperature.^1^ ^,^ ^14^ Instruments containing large amounts of martensite can be easily deformed, yet they will recover their shape on heating above the transformation temperature. In addition, NiTi alloys present remarkable flexural fatigue resistance in the martensitic state.^13^ ^,^ ^17^ Although VB instruments are not claimed to be produced with the CM technology, they also exhibit partial characteristic of shape memory at room temperature.^2^ ^,^ ^8^

The cyclic flexural fatigue resistance of an instrument is determined by its Nf values. As mentioned before, instrument diameter (D) and radius of curvature of the root canal (R) have been identified as the most important factors controlling fatigue resistance of endodontic instruments.^15^ ^,^ ^18^ ^-^ ^20^ The critical parameter is the maximum tensile strain amplitude, ε_T_, which is given by the expression ε_T_=D/(2R–D), when the canal radius is measured at the outer canal wall, as was done in this study. Considering the mean diameters at 3 mm from the tip (D3) of the instruments were the same as the artificial canal geometric standards, the differences in fatigue resistance of the instruments cannot be attributed only by the geometry, being the particularities in manufacturing process an influential factor. According to the literature, CM instruments exhibited a superior fatigue performance than instruments conventionally produced.^1^ ^,^ ^10^ ^,^ ^14^ Among the thermal treated instruments, HF presented a higher statistical cyclic flexural fatigue resistance, in comparison with TYP and VB.^7^ ^,^ ^14^ During root canal enlargement, each time the rotary NiTi instrument meets resistance, it undergoes torsional loading. The load is higher whenever the dentine is hard and the canal diameter is small. Acting on the instrument surface, this torsional load can prevent its rotation to a greater or lesser extent. Although this is the principle by which dentine can be cut, in extreme cases, when the resistance is so high that it constrains the instrument, it may fracture.^21^ As it was appreciated, VB instruments exhibited the highest torsional resistance in comparison with the other instruments, probably explained by a larger geometric factor such as A3 value.

Cyclic flexural fatigue and torsional resistance of NiTi files have been extensively evaluated as isolated processes.^9^ ^,^ ^10^ ^,^ ^14^ ^,^ ^22^ Some studies had correlated these two mechanisms together for superelastic files.^11^ ^,^ ^12^ ^,^ ^22^ ^,^ ^23^. However, only a few reports assessing these factors at the same time are available for CM instruments.^25^ ^-^ ^27^

In this study, a specific torsional loading cycle was previously applied to rotary instruments before their final rupture through cyclic flexural fatigue testing. The number of cycles employed in the load-unload tests was assumed to be roughly equivalent to the use of the instrument in half of the recommended number of uses for rotary instruments.^12^ Even though thermally treated files usually display a significantly higher cyclic flexural fatigue resistance than SE instruments, torsional preloads were able to significantly decrease the cyclic flexural fatigue resistance of the analyzed files.^2^ ^,^ ^14^ Furthermore, the results seem to indicate that heat treatments applied to HF, TYP, and VB files are not equivalent. Apparently, the same factors that increased cyclic flexural fatigue resistance in HF instruments acted to minimize torsional loading effects on them.

A remaining deformation was present in all instruments after unloading. This unrecovered strain was measured by means of the angular deflection values remaining when the torque reaches zero after unloading. For both CM instruments (HF and TYP) this angular deflection was similar. The behavior of VB instruments was statistically different with angular deflection after unloading reaching a lower value. This indicates that VB instruments have a partial superelastic behavior. This result agrees with the apparently higher slope of the VB loading curves (Figure 2C), which should reflect a larger amount of austenite in VB instruments.

The SEM images of the files fractured after torsional preloading and fatigued until rupture exhibited typical features of cyclic flexural fatigue resistance mode. Larger smooth areas of crack initiation, and slow propagation besides smaller dimple regions of final ductile fracture could be observed in the CM instruments in comparison with VB (Figure 3A-F), agreeing with previous studies.^1^ ^,^ ^14^ ^,^ ^25^ The images of the longitudinal view of the instruments after exposure to torsional loads displayed interesting characteristics. CM instruments showed fragmentation of the oxide layer developed under thermal treatment, while VB instruments presented a significant number of longitudinal cracks, which reflect the orientation of the stress on the surface of the instruments under torsional load. These cracks have been observed previously in superelastic instruments.^12^ ^,^ ^28^ but not in VB instruments. It is noteworthy that these longitudinal cracks did not interfere with the fatigue resistance of VB instruments, because the average decrease in Nf recorded for them (39%) was smaller than that geometrically similar to TYP instruments (45%), which did not develop longitudinal cracks. On the other hand, VB instruments showed a shape recover larger than the CM instruments after torsional loading, indicating that these instruments still conserve some characteristics of the conventional superelastic NiTi.

## Conclusions

Within the limitations of the study, the conclusion is that torsional preloading decreases the resistance to cyclic flexural fatigue of thermal treated instruments. In addition, Vortex Blue instruments exhibited some controlled memory behavior, displaying intermediate characteristics among CM instruments.
